# The Photocatalytic Efficacy of Potassium Hydroxide–Based Modification of Titanium Dioxide in the Oxidative Destruction of Gaseous Formaldehyde

**DOI:** 10.1002/smll.202501387

**Published:** 2025-04-17

**Authors:** Myeon‐Seong Cho, Kumar Vikrant, Danil W. Boukhvalov, Ki‐Hyun Kim

**Affiliations:** ^1^ Department of Civil and Environmental Engineering Hanyang University 222 Wangsimni‐Ro Seoul 04763 Republic of Korea; ^2^ College of Science Institute of Materials Physics and Chemistry Nanjing Forestry University Nanjing 210037 China; ^3^ Institute of Physics and Technology Ural Federal University Mira Street 19 Yekaterinburg 620002 Russia

**Keywords:** formaldehyde, indoor air, photocatalysis, potassium hydroxide, titanium dioxide

## Abstract

Formaldehyde (FA) is a carcinogenic oxygenated volatile organic compound and a key constituent of indoor air pollution. Photocatalytic oxidation (PCO) is a promising strategy for managing FA in indoor environments. Here, the PCO of FA in indoor air has been investigated by modifying TiO_2_ with KOH (KOH/TiO_2_: expressed as KT‐x, where x = the molar concentration of KOH from 0.1–2 m). The KOH treatment achieves superior FA removal performance of KT‐0.1 over TiO_2_ (e.g., space‐time yield: 1.78E−03 versus 9.91E−04 molecules photon^−1 ^mg^−1^ and dynamic clean air delivery rate: 10 versus 5.43 L mg^−1^ min^−1^). Such differences in photocatalytic activity reflect an enhancement in charge separation, molecular oxygen activation (on the photocatalyst surface), and electron mobility facilitated by the presence of surface hydroxyl groups and potassium on the TiO_2_ surface. On the KT‐x surface, the PCO of FA proceeds through several reactive intermediates (e.g., formate and dioxymethylene). According to density functional theory, the PCO of FA by KT‐x is promoted by the synergistic combination of oxygen vacancies and potassium impurities on the TiO_2_ surface. This research offers valuable insights into the development of cost‐effective photocatalysts with enhanced PCO performance.

## Graphical abstract

The photocatalytic oxidation of gaseous formaldehyde (FA) is explored using potassium hydroxide ‐modified titanium dioxide (TiO_2_), designated as KT‐x. Its superior performance is confirmed through dynamic clean air delivery rate and space‐time yield. The enhanced FA degradation over KT‐x is attributed to the synergistic effects of oxygen vacancies and potassium impurities on the TiO_2_ surface.

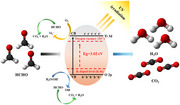

## 1 Introduction

Formaldehyde (FA), an oxygenated volatile organic compound (VOC), is commonly present in both outdoor and indoor environments. FA can be liberated to the atmosphere through industrial emissions and degassing from consumer products and materials, building materials, and furniture.^[^ ^1^, ^2^, ^3^, ^4^ ^]^ Exposure to FA can pose diverse health risks, ranging from respiratory problems (e.g., asthma and bronchitis) and allergic reactions (e.g., skin rash, itching, and conjunctivitis) to potential long‐term side effects (e.g., central nervous system damage and increased cancer risk).^[^ ^5^, ^6^ ^]^ FA is a Group 1 carcinogen, as defined by the International Agency for Research on Cancer.^[^ ^7^ ^]^ The US National Institute for Occupational Safety and Health has set a recommended exposure limit of 0.016 ppm (10 h time‐weighted average) for FA.^[^ ^8^ ^]^ As a result, an effective abatement technology against airborne FA is needed.

Multiple technologies have been deployed to mitigate the harmful effects of VOCs, such as adsorption, plasma catalytic oxidation, plant‐based purification, thermal catalytic oxidation, and photocatalysis.^[^ ^9^ ^]^ Among such options, photocatalytic oxidation (PCO) is the most attractive due to its efficiency, low environmental impact, and ability to operate under ambient conditions.^[^ ^10^ ^]^ PCO can decompose FA into environmentally benign carbon dioxide (CO_2_) and water (H_2_O) under ambient conditions. Titanium dioxide (TiO_2_) is among the most commonly used photocatalysts because of its non‐toxic nature, environmental compatibility, chemical stability, and affordability.^[^ ^11^, ^12^, ^13^ ^]^ However, the utility of TiO_2_ is limited by several drawbacks, such as a wide energy band gap of 3.2 eV (to absorb primarily ultraviolet [UV] light), rapid charge‐carrier recombination rate, low quantum efficiency, poor selectivity, photocorrosion, and aggregation in suspension.^[^ ^14^, ^15^, ^16^, ^17^ ^]^ Technical strategies have been proposed to lower the charge‐carrier recombination rate and/or reduce the band gap (e.g., element doping, metal deposition, and formation of heterojunctions).^[^ ^18^, ^19^, ^20^, ^21^, ^22^, ^23^ ^]^

Here, we describe a simple method of modifying TiO_2_ with potassium hydroxide (KOH) to enhance the oxidative removal of FA from indoor air under UV light irradiation. The process can be scaled up to be suitable for practical applications. The presence of potassium improves the photocatalyst performance by creating alternative pathways to facilitate electron mobility, enhancing charge‐separation efficiency, and promoting the formation of superoxide (˙O_2_^−^) and hydroxyl (^·^OH) radicals.^[^ ^24^, ^25^, ^26^ ^]^ FA oxidation can be mediated by directly oxidizing formate (e.g., a key intermediate during FA oxidation) into CO_2_.^[^ ^27^, ^28^ ^]^ Although there have been a few previous studies on KOH/TiO_2_, efforts to improve the PCO potential against FA or other VOCs in the air have been largely unsuccessful. This study offers deeper insights into the development of cost‐effective, practical photocatalysis of FA in indoor air under practical conditions.

## 2 Results and Discussion

### 2.1 Physical Properties of the Photocatalysts

#### 2.1.1 PXRD Patterns

**Figure 1:**
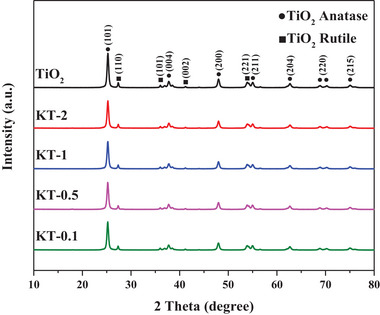
PXRD patterns of the tested photocatalysts.

Patterns of the tested photocatalysts revealed by PXRD are shown in **Figure** **1**. The peaks of KT‐x in comparison to those of TiO_2_ exhibited a gradual decrease in intensity with increasing KOH concentration, aligning with previous observations on the treatment of anatase TiO_2_ with KOH.^[^ ^29^ ^]^ No new peaks (e.g., those for potassium titanate) were present in the PXRD patterns for KT‐x, indicating that the inherent crystal structure remained unaltered after KOH treatment. The average anatase (101) and rutile (110) crystallite sizes were determined for all photocatalysts using the Scherrer equation (Equation 1; Table S1, Supporting Information).^[^ ^30^ ^]^ The average crystallite size decreased with increasing KOH concentration during KT‐x synthesis, likely due to the lower degree of TiO_2_ crystallization. Smaller average crystallite sizes increase surface and internal defects, increasing charge recombination and inhibiting photocatalytic activity.^[^ ^31^ ^]^ Controlling the average crystallite size of TiO_2_ is crucial to optimize photocatalytic activity:

(1) $$\mathrm{Average}\;\mathrm{crystallite}\;\mathrm{size}\;=\frac{K\lambda }{\beta cos\theta }$$

where K is the Scherrer constant (a value of 0.94 is used for spherical particles), β represents the full width at half maximum, λ represents the wavelength of the X‐ray (0.1541 nm for Cu Kα), and θ indicates the angle of diffraction.

#### 2.1.2 Surface Morphologies

**Figure 2:**
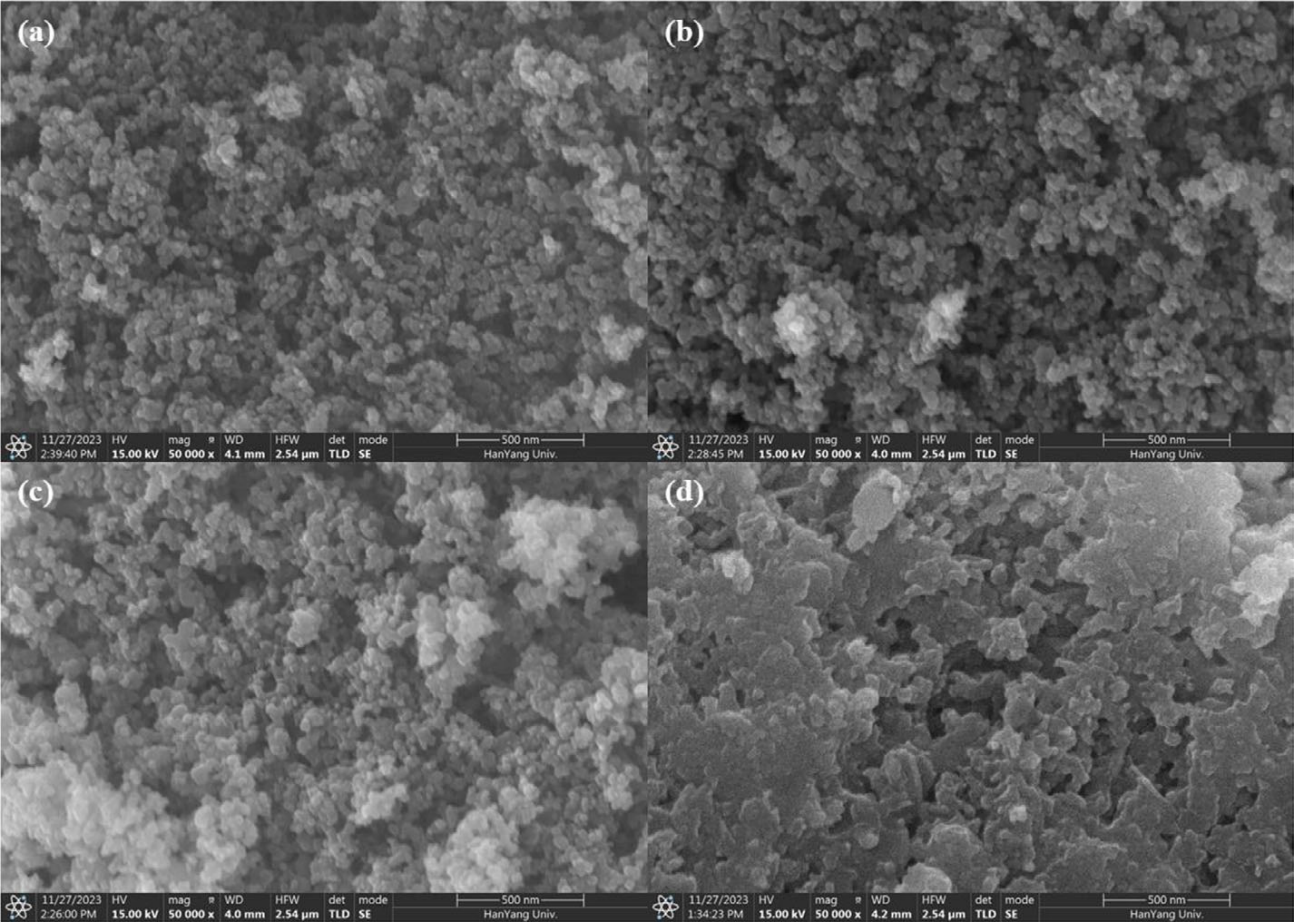
SEM images of the KT‐x photocatalysts: a) KT‐0.1, b) KT‐0.5, c) KT‐1, and d) KT‐2.

Slight differences were observed in the morphologies of the KT‐x photocatalysts, as seen from their SEM images (**Figure** **2**). The surface morphology changed with the KOH concentration to form diverse surface structures and crystals of various sizes and shapes. Specifically, KT‐0.1 and KT‐0.5 exhibited uniform and less agglomerated particles reminiscent of TiO_2_, as they were treated with low concentrations of KOH during synthesis, as shown in Figure 2a,b. In contrast, photocatalysts treated with higher KOH concentrations (i.e., KT‐1 and KT‐2) revealed agglomerated particles, as depicted in Figure 2c,d. In particular, KT‐2 exhibited a layered structure with significant agglomeration to reflect the effects of high KOH concentration on the oxide surface (Figure 2d). High alkaline conditions may lead to structural rearrangement and recrystallization through partial dissolution of oxides. This process is expected to induce a layered morphology through selective etching by KOH and surface reconstruction in a highly alkaline environment.^[^ ^32^, ^33^ ^]^

#### 2.1.3 N_2_ Adsorption–Desorption Isotherms

**Figure 3:**
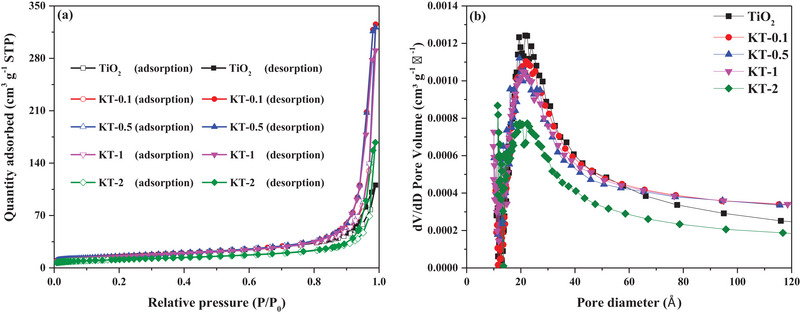
Surface properties of the tested photocatalysts. a) N_2_ adsorption‐desorption isotherms and b) pore‐size distribution curves.

**Figure** **3**a shows the N_2_ adsorption–desorption isotherms for the tested photocatalysts. A type‐IV character with H3 hysteresis loops was displayed by the isotherms for all tested photocatalysts per the International Union of Pure and Applied Chemistry database, indicating the predominant mesoporosity of the samples.^[^ ^34^ ^]^ Based on the Barrett–Joyner–Halenda model and as shown in Figure 3b, the pores were distributed in the 1.5‐to‐10 nm range. The Brunauer–Emmett–Teller (BET) surface area, average pore diameter, and pore volume of the tested photocatalysts are listed in Table S1 (Supporting Information). The BET surface area of the tested photocatalysts decreased in the following order: TiO_2_ (50.9 m^2^ g^−1^) > KT‐0.1 (50.5 m^2^ g^−1^) > KT‐0.5 (49.6 m^2^ g^−1^) > KT‐1 (49.1 m^2^ g^−1^) > KT‐2 (38.8 m^2^ g^−1^), indicating that KOH modification decreased the surface area. The pore volume (cm^3^ g^−1^) followed the order of KT‐0.1 (0.077) > KT‐0.5 (0.076) > TiO_2_ (0.068) > KT‐1 (0.063) > KT‐2 (0.049), a trend similar to that of FA adsorption (Section 3.4.1). The average pore diameter followed the order of KT‐0.1 (4.99 nm) > KT‐0.5 (4.84 nm) > KT‐1 (4.72 nm) > TiO_2_ (4.44 nm) > KT‐2 (4.31 nm). The results suggest that higher KOH concentrations should lead to the aggregation of TiO₂ particles and alterations in their pore structures (e.g., partial blockage or merging). The occurrences of such structural modifications are supported by apparent reductions in pore volume (from 0.068 to 0.049 cm^3^ g^−1^) and in average pore diameter (from 4.44 to 4.31 nm). It thus suggests that surface restructuring intensified with increasing KOH concentration, particularly in KT‐2, where the most significant decrease was observed.

### 2.2 Surface Chemistry

**Figure 4:**
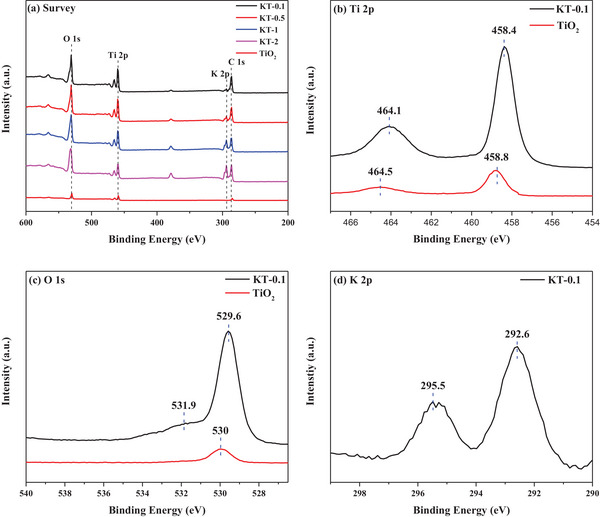
XPS spectra of KT‐x and TiO_2_: a) Survey, b) Ti 2p, c) O 1s, and d) K 2p.

The XPS survey spectrum exhibited C 1s, Ti 2p, O 1s, and K 2p signals for both KT‐x and TiO_2_ (**Figure** **4**a). The at.% values of each species on the photocatalyst surface are summarized in Table S2 (Supporting Information). Figure 4b shows the deconvoluted Ti 2p KT‐0.1 spectrum, with peaks for Ti 2p_3/2_ and Ti 2p_1/2_ at 458.4 and 464.1 eV, respectively. The redshift of the KT‐0.1 Ti 2p_3/2_ peak (e.g., by −0.4 eV relative to that for TiO_2_) indicates that Ti^4+^ should partially be reduced to Ti^3+^ by the KOH treatment. In general, Ti^3+^ formation is closely related to the creation of an oxygen vacancy (OV). As such its presence suggests an interaction between potassium ions and the TiO_2_ surface.^[^ ^35^, ^36^ ^]^ The peak at 529.6 eV in the deconvoluted KT‐0.1 O 1s spectrum was attributed to the Ti─O bond, as depicted in Figure 4c. Also, compared to TiO_2_, the shoulder peak at 531.9 eV in the deconvoluted KT‐0.1 O 1s spectrum indicated surface‐adsorbed OH.^[^ ^37^ ^]^ The Ti─O peak in the deconvoluted KT‐0.1 O 1s spectrum showed a −0.4 eV redshift compared to TiO_2_, indicating a change in the oxygen environment around titanium associated with the partial reduction of Ti^4+^ to Ti^3+^.^[^ ^38^ ^]^ The deconvoluted KT‐0.1 K 2p spectrum displayed K 2p_1/2_ and K 2p_3/2_ peaks at 292.6 and 295.5 eV, respectively, aligning with previous results as shown in Figure 4d.^[^ ^39^ ^]^ This suggests that the interaction between KOH and TiO₂ follows a multifaceted mechanism. Potassium ions likely coordinate with surface oxygen atoms, while hydroxide ions adsorb onto the TiO₂ surface. Additionally, the direct bonding of potassium ions to Ti atoms and the generation of oxygen vacancies (OVs) during KOH treatment play crucial roles in this process. This analysis supports the hypothesis that KOH attaches to TiO₂ through a combination of ionic interactions, OH adsorption, and structural modifications, including the reduction of Ti⁴⁺ to Ti^3^⁺ and the formation of oxygen vacancies (OVs).

### 2.3 Raman Spectroscopy

**Figure 5:**
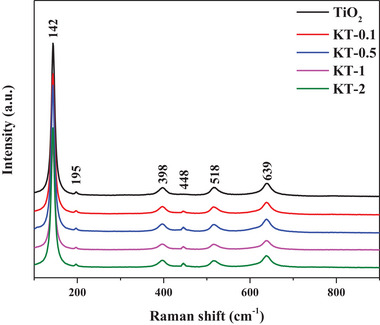
Raman spectra of KT‐x and TiO_2_.

**Figure** **5** shows the Raman spectra for KT‐x and TiO_2_ with the characteristic bands observed at 142, 195, 398, 517, and 638 cm^−1^. These bands correspond to the Raman‐active modes of the anatase phase of TiO_2_, namely E_g_ (142 cm^−1^), E_g_ (195 cm^−1^), B_1_ _g_ (398 cm^−1^), A_1_ _g_ or B_1_ _g_ (517 cm^−1^), and E_g_ (638 cm^−1^) modes.^[^ ^40^, ^41^, ^42^ ^]^ In particular, the Eg mode at 142 cm⁻¹, attributed to the O─Ti─O symmetric stretching vibration, exhibited the strongest signal. Additionally, a new band at 448 cm⁻¹, absent in pristine TiO₂, appeared in all KT‐x samples. This peak should be attributed to structural modifications induced by potassium incorporation, which likely alters the TiO₂ crystal lattice.^[^ ^39^ ^]^ The consistent presence of the 448 cm⁻¹ band across all KT‐x samples further indicates that the structural modification should have occurred uniformly to enhance catalytic or photocatalytic performance with the abundant active sites and enhanced electron mobility.

### 2.4 Photoelectric Properties

**Figure 6:**
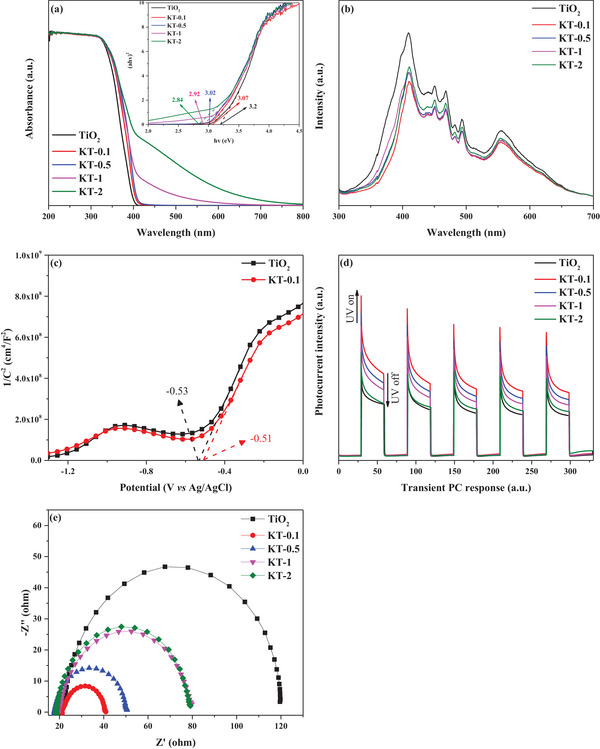
Photoelectric properties of the tested photocatalysts: a) UV–vis DRS spectra. Inset: Tauc plots. b) PL spectra. c) MS plot. d) Transient PC response under UV on/off cycles. e) EIS spectra.

The light‐absorption characteristics of TiO_2_ and KT‐x were investigated using UV–vis DRS. As shown in **Figure** **6**a, the KT‐x light‐absorption tendency improved (compared to TiO_2_) with KOH concentration during KT‐x synthesis. The KT‐x samples also displayed redshifts in the TiO_2_ light‐absorption edge. The bandgap energies of the tested photocatalysts were estimated using the Kubelka–Munk function.^[^ ^43^ ^]^ The band gap of TiO_2_ was consistent with that reported in the literature (3.2 eV). In contrast, the band gap values of KT‐x exhibited a gradually decreasing trend such as 3.07, 3.02, 2.92, and 2.84 eV with increases in KOH concentrations for the modifications (0.1, 0.5, 1, and 2 m, respectively). These reductions in band gap can be attributed to potassium on the surface of TiO_2_.^[^ ^44^ ^]^ Figure 6b shows the photoluminescence spectra of the tested photocatalysts. A lower peak intensity in the PL spectrum indicates superior photocatalysis.^[^ ^45^ ^]^ In this regard, the KT‐x samples for which the lowest KOH concentrations were used during synthesis displayed the smallest peaks (likely due to enhanced photogenerated electron‐hole pair separation) compared with TiO_2_. An MS analysis was conducted to investigate the electrochemical properties of TiO_2_ and KT‐0.1. Specifically, it was used to obtain the donor density, flat band potential, and conduction band (Equations 2 and 3):

(2) $$\frac{1}{C^{2}}=\frac{2}{\varepsilon \times \varepsilon_{0}\times e\times N_{D}}\times \left(V-V_{FB}-\frac{k_{B}T}{e}\right)$$

(3) $$N_{D}=\frac{2}{\varepsilon \times \varepsilon_{0}\times e}\times \frac{1}{slope}$$

where ɛ represents the dielectric constant, C (cm^4^ F^−2^) indicates the space charge capacitance of the electrolyte‐n‐type film interface, ɛ_0_ represents vacuum permittivity (8.86 × 10⁻¹⁴ F cm⁻¹), N_D_ (cm^−3^) indicates the n‐type semiconductor donor density, e represents the elementary charge of an electron (1.6 × 10^−19^ C), V indicates the applied potential (as a reference to V_Ag/AgCl_), k_B_ represents the Boltzmann constant (1.38 × 10^−23^ J K^−1^), V_FB_ indicates the flat band potential (referenced to V_Ag/AgCl_), and T represents the absolute temperature (K). Positive slopes were observed in the MS plots (relationship between 1/C^2^ and band potential) of KT‐0.1 and TiO_2_ in Figure 6c to indicate that these materials exhibit n‐type semiconductor characteristics. According to Equation 2, the slope of the KT‐0.1 MS plot (2.12 × 10^9^) was smaller than that of TiO_2_ (2.11 × 10^9^). In addition, the plateau potential (V_FB_) of KT‐0.1 was −0.51 eV, which is more negative than that of TiO_2_ (−0.53 eV), likely due to the presence of Ti^3+^ and OVs in the former. Using the equation E_NHE_ = E_Ag/AgCl_ + 0.1976 (298 K), the normal hydrogen electrode (N_HE_) scale values for KT‐0.1 and TiO_2_ were computed as −0.31 and −0.33 eV, respectively, indicating stronger negative redox potentials than that of O_2_/^•^O_2_^−^ (−0.18 V vs N_HE_), i.e., promotion of the one‐electron reduction of O_2_ and increased generation of ^•^O_2_^−^.^[^ ^46^ ^]^ According to Equation 3, the KT‐0.1 and TiO_2_ N_D_ values were 1.38 × 10^22^ and 1.43 × 10^22^ cm^−3^, respectively. A lower donor density means a wider depletion layer width, improving the charge‐separation efficiency.^[^ ^47^ ^]^

The transient PC indirectly reflects the ability of a photocatalyst to generate and transfer photogenerated charge carriers.^[^ ^48^ ^]^ Figure 6d shows the transient PC response of the tested photocatalysts to UV on/off cycles. The transient PC response intensity follows the order of KT‐0.1 > KT‐0.5 > KT‐1 > KT‐2 > TiO_2_, with KT‐0.1 exhibiting a PC intensity 2.05 times stronger than that of TiO_2_. This result indicates that the most efficient photogenerated electron‐hole pair separation occurs in KT‐0.1. Figure 6e presents EIS Nyquist plots for the tested photocatalysts. The reduction in arc radius becomes more pronounced as the KOH concentration during KT‐x synthesis decreases. The relatively small arc radius in the EIS spectrum of KT‐0.1 indicates improved separation efficiency of the photogenerated charge carriers.^[^ ^49^ ^]^ KOH treatment of TiO_2_ reduces charge‐transfer resistance and enhances photocatalytic activity by improving the photogenerated charge‐separation efficiency.

### 2.5 Photocatalytic FA Oxidation Performance

#### 2.5.1 Effect of the KOH:TiO_2_ Ratio and Light Source

**Figure 7:**
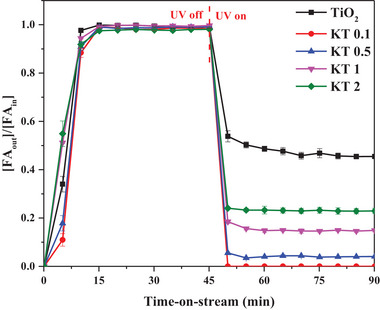
FA removal performance of the tested photocatalysts as a function of time‐on‐stream ([FA_in_]: 100 ppm in air, Q: 100 mL min^−1^, RH: 0%, m_cat_: 10 mg, and light source: UV‐A).

In the present study, the PCO performance of the synthesized photocatalysts (e.g., TiO_2_ and KT‐x) was investigated using a laboratory‐scale, continuous‐flow, packed‐bed reactor with FA (100 ppm) as the target VOC (**Figure** **7**). The tested materials behaved as adsorbents under the UV‐off condition, with the FA gaseous working standard (G‐WS) volume required to attain 50% breakthrough (BT 50%) decreasing in the following order: KT‐0.1 (0.76 L) > KT‐0.5 (0.73 L) > TiO_2_ (0.64 L) > KT‐1 (0.49 L) > KT‐2 (0.47 L). The KOH modification of TiO_2_ enhanced FA adsorption. Under illumination with UV light, all KT‐x photocatalysts showed improved FA degradation efficiency compared to TiO_2_. The steady‐state X_FA_ decreased in the following order: KT‐0.1 (100%) > KT‐0.5 (96.5%) > KT‐1 (85.5%) > KT‐2 (77.1%) > TiO_2_ (54.3%) (**Table** **1**a). The KT‐0.1 exhibited the best FA PCO performance among the tested photocatalysts, with a QY (1.78E−02 molecules photon^−1^) 1.8 times higher than that of TiO_2_ (9.91E−03 molecules photon^−1^). The enhancement in FA removal performance of KOH‐modified TiO_2_ can be attributed to the existence of potassium and OH groups on the surface. However, a high KOH concentration during KT‐x synthesis could have blocked the TiO_2_ pores, reducing the number of available surface sites and lowering the overall FA removal performance.^[^ ^29^ ^]^ To investigate the potential effects of KOH concentration on KT‐x performance, an additional experiment was conducted using samples with higher KOH concentrations, such as KT‐3 and KT‐5 (Figure S2, Supporting Information), as detailed in the supplementary information (SI). A gradual reduction in performance was observed, confirming the decrease in active surface sites with increasing KOH concentrations. Based on these findings, KT‐0.1 was selected as the best‐performing sample to analyze the effects of process variables on photocatalytic activity.

**Table 1:** FA PCO performance of KT‐x and TiO_2_ under diverse conditions.

Order	Catalyst	RH	[FA_in_]	Q	O_2_ content	X_FA_	DCADR	QY	STY
		(%)	(ppm)	(mL min^−1^)	(%)	(%)	(L mg^−1^ min^−1^)	(molecules photon^−1^)	(molecules photon^−1^ mg^−1^)
(a) Photocatalyst									
1	TiO_2_	0	100	100	21	54.3	5.43	9.91E‐03	9.91E‐04
2	KT‐0.1					100	10	1.78E‐02	1.78E‐03
3	KT‐0.5					96.5	9.65	1.72E‐02	1.72E‐03
4	KT‐1					85.5	8.55	1.52E‐02	1.52E‐03
5	KT‐2					77.1	7.71	1.37E‐02	1.37E‐03
(b) RH									
6	KT‐0.1	0	200	100	21	65.2	6.52	2.31E‐02	2.31E‐03
7		20				80.6	8.06	2.87E‐02	2.87E‐03
8		40				75.2	7.52	2.68E‐02	2.68E‐03
9		60				60.4	6.04	2.15E‐02	2.15E‐03
10		80				53.8	5.38	1.92E‐02	1.92E‐03
(c) [FA_in_]									
13	KT‐0.1	20	100	100	21	100	10	1.78E‐02	1.78E‐03
14			200			80.6	8.06	2.87E‐02	2.87E‐03
15			300			58.4	5.84	3.12E‐02	3.12E‐03
(d) Q									
16	KT‐0.1	20	100	100	21	100	10	1.78E‐02	1.78E‐03
17				200		82.6	16.52	2.94E‐02	2.94E‐03
18				300		55.3	16.59	2.96E‐02	2.96E‐03
(e) O_2_ content									
19	KT‐0.1	20	100	100	0	62.5	6.25	1.11E‐02	1.11E‐03
20					5	78.3	7.83	1.40E‐02	1.40E‐03
21					10	90.9	9.09	1.64E‐02	1.64E‐03
22					21	100	10	1.78E‐02	1.78E‐03
(f) Reusability									
23	KT‐0.1	20	100	100	21	100	10	1.78E‐02	1.78E‐03
24									
25									
26									

To learn more about the photocatalytic properties of the KT samples, their photocatalytic behavior was also studied under visible light illumination conditions (Figure S3, Supporting Information). Compared to UV irradiation, the X_FA_ values exhibited a noticeable drop (e.g., by an average of 25.2%). This reduction in photocatalytic activity can be attributed to the lower photon energy of visible light, which limits charge carrier excitation with the rapid electron‐hole recombination rate.

#### 2.5.2 Effects of Process Variables

**Figure 8:**
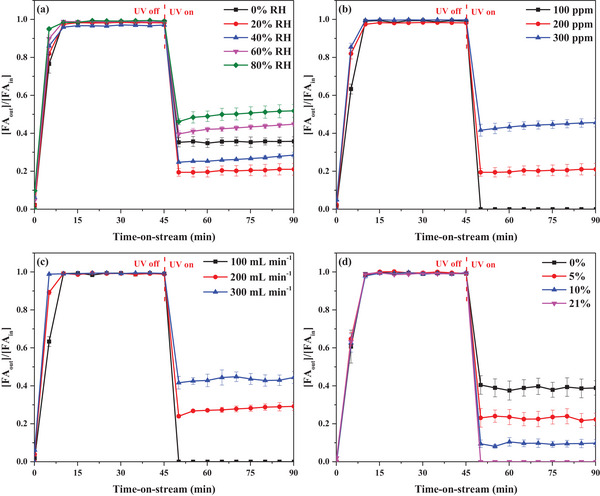
FA removal performance of KT‐0.1 as a function of time‐on‐stream and other process variables. a) RH ([FA_in_]: 200 ppm in air, Q: 100 mL min^1^, m_cat_: 10 mg, and light source: UV‐A). b) [FA_in_] (RH: 20%, Q: 100 mL min^−1^, m_cat_: 10 mg, and light source: UV‐A). c) Q ([FA_in_]: 100 ppm in air, RH: 20%, m_cat_: 10 mg, and light source: UV‐A). d) O_2_ content ([FA_in_]: 100 ppm in air, RH: 20%, Q: 100 mL min^−1^, m_cat_: 10 mg, and light source: UV‐A).

As moisture is ubiquitous in the air, it is critical to analyze the PCO activity as a function of RH. As KT‐0.1 achieved 100% removal of 100 ppm of FA under dry conditions, a 200 ppm G‐WS of FA was used to better visualize how moisture controls photocatalytic activity. Under the UV‐off condition, increased RH lowered the FA adsorption performance as the H_2_O and VOC molecules competed for active surface sites.^[^ ^50^, ^51^ ^]^ As shown in **Figure** **8**a, moisture promoted FA PCO, with a QY 1.24 times that of the dry condition. The decreasing order of QY (molecules photon^−1^) as a function of RH was as follows: 20% RH (2.87E−02) > 40% RH (2.68E−02) > 0% RH (2.31E−02) > 60% RH (2.15E−02) > 80% RH (1.92E−02). Details are provided in Table 1b. Moisture promoted PCO performance as a greater amount of OH was generated to facilitate FA oxidation. However, the H_2_O molecules compete with FA for active surface sites at higher RH levels, lowering the overall FA PCO performance. Also, an H_2_O film can form on the surface of a photocatalyst at elevated RH, lowering FA PCO performance by increasing the recombination rate of the photogenerated electron‐hole pairs and blocking reactant molecules from accessing the active sites on the surface.^[^ ^52^, ^53^ ^]^

The influence of an [FA_in_] of 100–300 ppm on the photocatalytic activity of KT‐0.1 at 20% RH was also investigated. The results are shown in Figure 8b. Under the UV‐off condition, the breakthrough became faster as the [FA_in_] increased. The X_FA_ decreased in the following order under UV irradiation: 100% (100 ppm) > 70.6% (200 ppm) > 58.4% (300 ppm). The QY (molecules photon^−1^) decreased in the reverse order, i.e., 300 ppm (3.12E−02) > 200 ppm (2.94E−02) > 100 ppm (1.78E−02). These findings suggest that the high reactivity of KT‐0.1 surface sites results in the rapid capture and decomposition of FA molecules during photocatalysis. Unlike X_FA_, QY increased with [FA_in_] as more FA molecules could be degraded per unit absorbed photons.

The Q exerts a tight control on the contact time between the reactants and the photocatalyst. In the adsorption stage, as depicted in Figure 8c, as Q increased from 100 to 300 mL min^−1^, the breakthrough accelerated due to decreased residence time, resulting in poor FA capture at the photocatalyst surface. The FA PCO performance in terms of X_FA_ exhibited the same trend as the adsorption stage: 100 mL min^−1^ (100%) > 200 mL min^−1^ (76.1%) > 300 mL min^−1^ (55.3%). In contrast, the QY decreased in the order of 300 mL min^−1^ (3.12E−02 molecules photon^−1^) > 200 mL min^−1^ (2.94E−02 molecules photon^−1^) > 100 mL min^−1^ (1.78E−02 molecules photon^−1^). The reverse trend for QY can be attributed to more FA molecules being degraded per absorbed photon due to increased space velocity. O_2_ plays a crucial role in FA PCO by generating ˙O_2_^−^. The FA adsorption performance in the UV‐off condition) was not affected significantly by the changing O_2_ concentration, as shown in Figure 8d. However, under UV irradiation, the PCO performance decreased in the order of 21% O_2_ (100%) > 10% O_2_ (95.6%) > 5% O_2_ (76.9%) > 0% O_2_ (59.6%). The same trend was evident in QY: 21% O_2_ (1.78E−02) > 10% O_2_ (1.64E−02) > 5% O_2_ (1.4E−02) > 0% O_2_ (1.11E−03). From the perspective of QY, 21% O_2_ achieved a performance 1.6 times that of 0% O_2_.

#### 2.5.3 Photocatalyst Reusability and FA Mineralization

**Figure 9:**
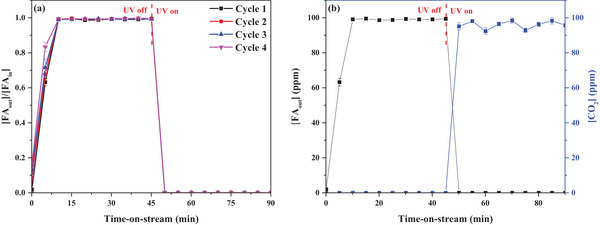
FA removal performance of KT‐0.1 as a function of time‐on‐stream. a) Photocatalyst reusability ([FA_in_]: 100 ppm in air, RH: 20%, Q: 100 mL min^−1^, and m_cat_: 10 mg). b) FA mineralization ([FA_in_]: 100 ppm in air, RH: 20%, Q: 100 mL min^−1^, and m_cat_: 10 mg).

One of the most critical considerations in applying photocatalysis is reusability. Reusability experiments can evaluate the stability and durability of photocatalysts. Reusable photocatalysts can dramatically reduce the cost and environmental impact of the process. As shown in **Figure** **9**a, the reusability of the photocatalyst was evaluated four times under optimal conditions. KT‐0.1 was evaluated for its adsorptive PCO performance over four cycles without further treatment. The FA molecules and reaction intermediates that accumulated during repeated adsorption and oxidation likely saturated and blocked the active surface sites, steadily decreasing adsorption performance across the test cycles under UV‐off conditions.^[^ ^54^ ^]^ However, under UV irradiation, 100% X_FA_ was maintained over four cycles, indicating the high potential of the tested photocatalyst for use in practical indoor air purification.

The Y_CO2_ was also measured for the first cycle of the reusability experiment to quantify the FA mineralization efficiency of KT‐0.1 As can be seen in Figure 9b, incomplete oxidation of FA likely generated hazardous by‐products. Complete mineralization of FA into CO_2_ is necessary to prevent secondary pollution and ensure environmental safety. Under UV‐off conditions, no FA was oxidized by KT‐0.1. However, under UV irradiation, CO_2_ generation was observed, indicating the occurrence of FA PCO. A maximum Y_CO2_ of 98.4% was attained. This suggests that KT‐0.1 can almost completely mineralize FA during PCO.

### 2.6 In Situ DRIFTS and ESR Analysis

**Figure 10:**
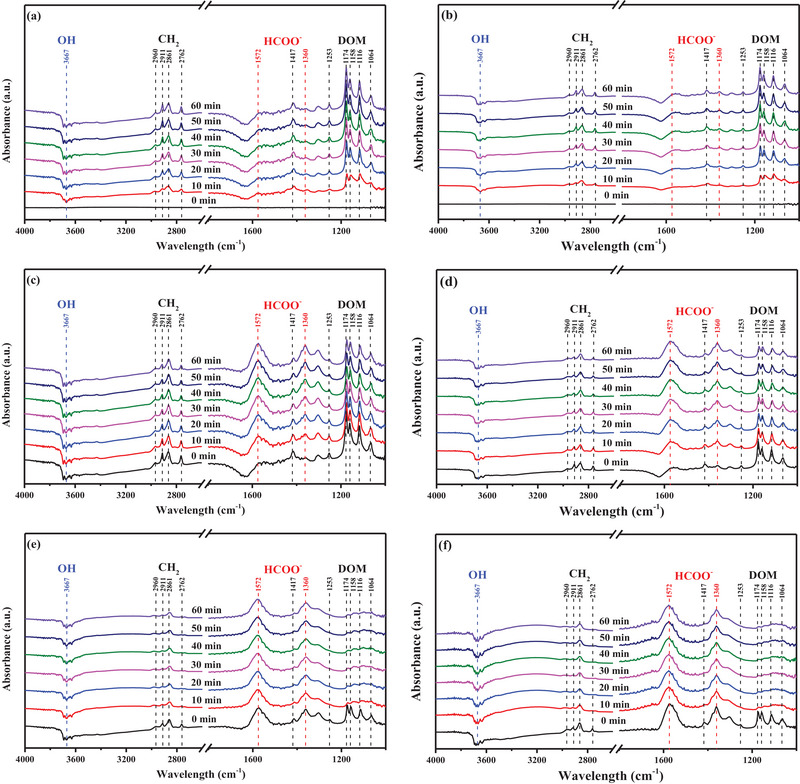
In situ DRFITS spectra under different reaction conditions: a) KT‐0.1 (FA + air + UV off), b) TiO_2_ (FA + air + UV off), c) KT‐0.1 (FA + air + UV on), d) TiO_2_ (FA + air + UV on), e) KT‐0.1 (FA + air + H_2_O + UV on), f) TiO_2_ (FA + air + H_2_O + UV on).

In situ DRIFTS analysis was used to assess the reaction pathway for FA oxidation on the KT‐0.1 and TiO_2_ surfaces. All analyses were performed under air (21% O_2_) flow with 100 ppm FA. **Figure** **10**a,b depict the in situ DRIFTS spectra of respective KT‐0.1 and TiO_2_ under dark conditions for a dry gas flow (RH = 0%). Bands at 1064, 1116, 1158, 1174, and 1253 cm^−1^ originated from the ν(CO) of dioxymethylene (DOM). The bands at 1417 cm^−1^ belonged to the δ(CH_2_) of DOM, while the bands at 2762, 2861, 2911, and 2960 cm^−1^ were ascribed to the ν(CH_2_) of DOM.^[^ ^55^ ^]^ The bands at 1360 and 1572 cm^−1^ were associated with symmetric (ν_s_(COO^−^)) and asymmetric (ν_as_(COO^−^)) formate stretching, respectively.^[^ ^56^ ^]^ A negative absorbance band for surface OH near 3667 cm^−1^ indicates its consumption during FA adsorption and oxidation.^[^ ^57^ ^]^

Figure 10c,d depicts the in situ DRIFTS spectra of KT‐0.1 and TiO_2_, respectively, under UV irradiation for a dry gas flow (RH = 0%). The DOM band intensity progressively decreased under UV irradiation as it converted to formate.^[^ ^58^ ^]^ KT‐0.1 showed higher DOM band intensity than TiO_2_ as it had more active surface sites for FA adsorption and reaction, as shown in Figure S4b (Supporting Information). Formate tended to gradually accumulate over time due to the conversion of DOM to formate.^[^ ^59^ ^]^ KT‐0.1 exhibited a lower OH band intensity compared with that of TiO_2_ due to its higher photocatalytic activity (efficient consumption of surface OH groups), as shown in Figure S4a (Supporting Information).^[^ ^60^ ^]^

Figure 10e,f depicts the in situ DRIFTS spectra (KT‐0.1 and TiO_2_) under UV irradiation for a humid gas flow (RH = 20%). The OH band intensity became more prominent for KT‐0.1 and TiO_2_ in a humid environment, indicating the formation of surface OH groups. The use of KT‐0.1 produced a relatively low accumulation of surface OH due to its efficient consumption during FA oxidation, as shown in Figure S4a (Supporting Information). The DOM bands were significantly attenuated under humid conditions due to their transformation into FA oxidation products through interactions with ROS. KT‐0.1 produced a lower DOM band intensity compared with TiO_2_, as the former generated ROS more efficiently, as in Figure S4b (Supporting Information).^[^ ^61^ ^]^ The introduction of moisture into KT‐0.1 led to the rapid conversion of FA into DOM, resulting in the accumulation of formate. Consequently, the accumulation of formate on the photocatalyst surface was stabilized at a constant level as it was rapidly transformed into FA mineralization products (Figure S4c, Supporting Information).

**Figure 11:**
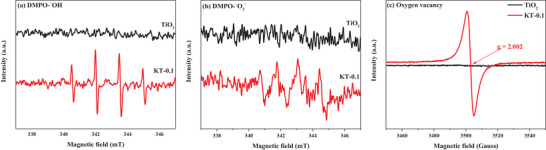
ESR/electron paramagnetic resonance (EPR) spectra for KT‐0.1 and TiO_2_. a) DMPO‐˙OH, b) DMPO‐˙O_2_^−^, and c) OV.

The ESR spin‐trap technique with a DMPO adduct was used to investigate the generation of ROS by KT‐0.1 and TiO_2_. In **Figure** **11**, the presence of ˙O_2_^−^ and ˙OH in KT‐0.1 and TiO_2_ under UV irradiation was confirmed. In Figure 11a, the ESR spectrum of KT‐0.1 showed stronger DMPO‐˙OH quadruple signals (340.5, 341.9, 343.5, and 344.9 mT) than TiO_2_. Similarly, the DMPO‐˙O_2_^−^ signals were also much stronger for KT‐0.1 (340.7, 341.8, 343.1, and 344.4 mT) than TiO_2_. These results indicate the superior photocatalytic activity of KT‐0.1 to TiO_2_. In addition to the enhanced ROS production, Figure 11c confirms the presence of OVs in KT‐0.1. The signal at g = 2.002, attributed to OVs and trapped electrons, is significantly stronger in KT‐0.1 than in TiO_2_. This indicates that KOH treatment effectively forms OVs in TiO_2_, which serve as active sites to enhance charge separation efficiency and to improve photocatalytic activity.

### 2.7 DFT Simulation

**Figure 12:**
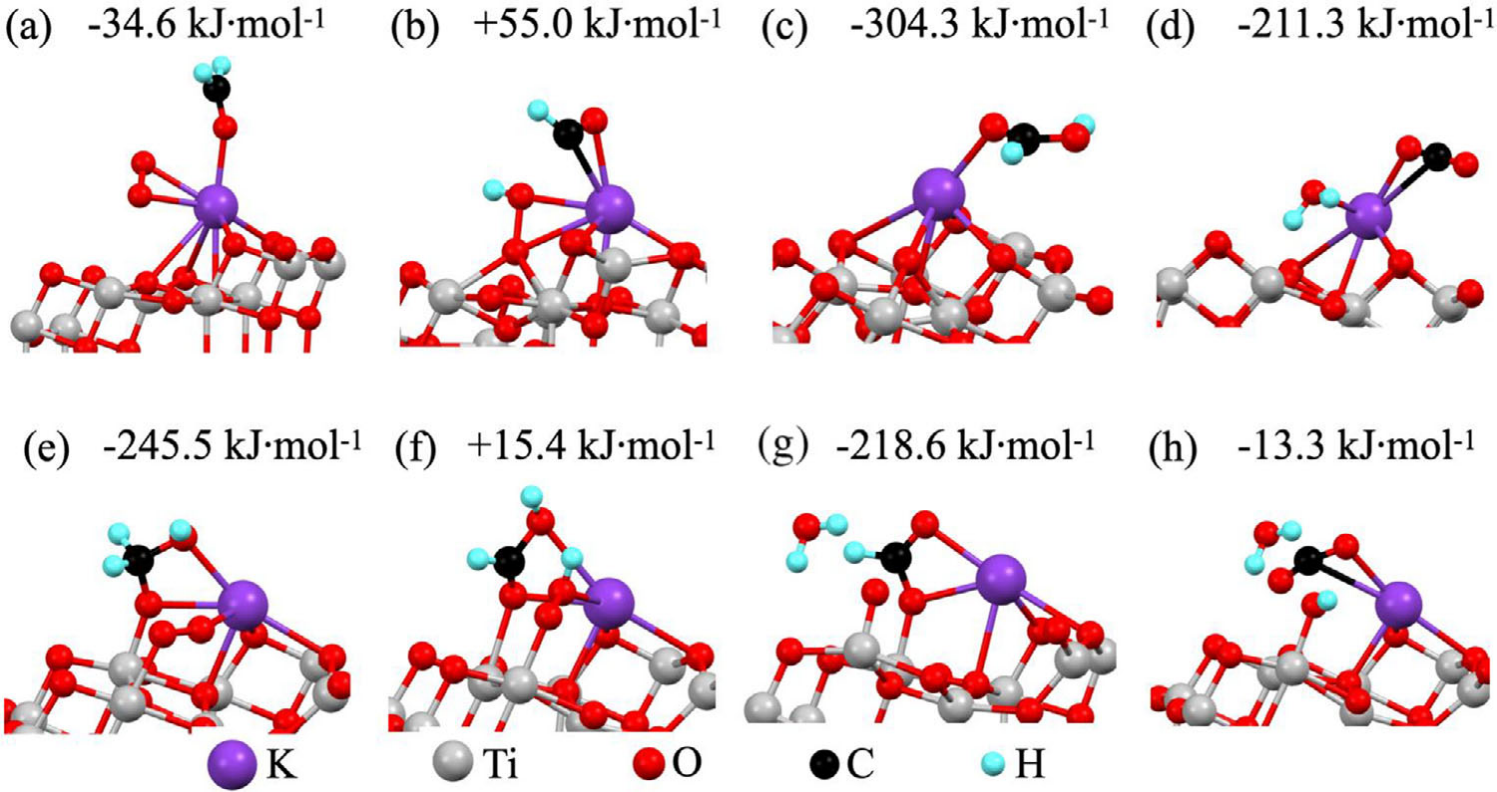
Optimized atomic structures and the corresponding energies for FA conversion steps on the tested surfaces: a–d) represent the TiO_2_ (101) surface with a single potassium atom, while e–h) represent the TiO_2_ (101) surface with K‐OH near OVs.

A slab of 96 atoms corresponding to the (101) surface of anatase TiO_2_ was used to simulate FA conversion, as described previously (Wang et al., 2024; Zhang et al., 2024). A TiO_2_ surface with OVs in the outermost layer was also simulated. Single impurities incorporated into the surface were simulated based on XPS analysis. DFT calculations showed that the formation of a single interstitial impurity was 300 kJ mol^−1^ more favorable than that of the substitutional impurity (**Figure** **12**). The K─OH embedded in the TiO_2_ surface with and without OVs was also simulated to study the impact of ─OH adsorbed on the potassium center (Figure 12e–i). The first simulation step was the free‐energy calculation for O_2_ and FA adsorption on the four substrates, as shown in Figure 12a,e, and was exothermic for all simulated surfaces. The second step involved hydrogen migration (from the FA molecule to surface‐adsorbed O_2_) by conversion of O_2_ into ─OOH (covalently adsorbed on the substrate), as shown in Figure 12b,f. This step was also exothermic, with an energy gain greater than 100 kJ mol^−1^ for the potassium center embedded in TiO_2_ near OV or K─OH in the substrate without OVs. The subsequent conversion steps over these surfaces were also exothermic, with greater than 100 kJ mol^−1^ energy gain.

The conversion of FA leads to stable structures that eventually lead to the deactivation of potassium centers (Figure S5, Supporting Information). Such deactivation in oxygen‐rich TiO_2_ and K─OH near OVs was endothermic, with a moderate energy cost of less than 60 kJ mol^−1^. The subsequent steps of FA conversion were exothermic, as illustrated in Figure 12c,d,h,i. The final steps of FA conversion corresponded to the formation of H_2_O and CO_2_, which were physisorbed onto the substrate, as shown in Figure 12d,i. The desorption of these molecules was endothermic, with energies of 37.2 and 44 kJ mol^−1^, respectively, and reactivated the catalysts. Based on the results of the simulations, the difference in catalytic performance of the tested samples can be accounted for by potassium‐based surface impurities and OVs. For instance, excessive KOH treatment (and high K loading) can passivate active sites, leading to catalytic deactivation due to surface coverage and structural changes (Figure S5, Supporting Information). Although stable but inactive sites ensure long‐term durability, they may require additional modifications (e.g., co‐catalyst loading) to restore activity. Thus, proper control on potassium loading should be crucial to balancing activity and stability in KOH‐treated TiO₂.

### 2.8 FA PCO Performance Comparison

The FA PCO performance of KT‐0.1 was compared with those of previously reported photocatalysts used in continuous‐flow systems (**Table** **2**). KT‐0.1 achieved outstanding QY/STY performance with 300 ppm FA at 20% RH and a Q of 100 mL min^−1^. KT‐0.1 (3.12E−02 molecules photon^−1^) was the most efficient photocatalyst for FA removal, followed by gold/TiO_2_ (1.39E−03 molecules photon^−1^) > copper‐TiO_2_ (3.88E−06 molecules photon^−1^) > platinum/TiO_2_ nanowire (6.16E−07 molecules photon^−1^) > TiO_2_ nanowire (5.61E−07 molecules photon^−1^) > TiO_2‐x_‐OV (4.96E−08 molecules photon^−1^). The STY performance (molecules photon^−1^ mg^−1^) followed the order of KT‐0.1 (3.12E−03) > Au/TiO_2_ (9.24E−05) > Pt/TiO_2_ nanowire (6.16E−08) > Cu‐TiO_2_ (3.88E−08) > TiO_2_ nanowire (1.12E−08) > TiO_2‐x_‐OV (4.96E‐10). These results suggest the superiority of KT‐0.1 for PCO of FA.

**Table 2:** FA PCO performance comparison between KT‐0.1 and other catalysts.

Order	Photocatalyst	RH	Light power	λ_max_	Q	m_cat_	[FA_in_]	X_FA_	QY	STY	Refs.
		(%)	(W)	(nm)	(L min^−1^)	(mg)	(ppm)	(%)	(molecules photon^−1^)	(molecules photon^−1^ mg^−1^)	
1	Au/TiO_2_	16	1	365	0.2	15	50	62	1.39E−03	9.24E−05	[71]
2	Cu‐TiO_2_	50	500	420	0.1	100	100	100	3.88E−06	3.88E−08	[72]
3	Pt/TiO_2_ nanowire	80	6	420	0.05	10	0.41	99	6.16E−07	6.16E−08	[73]
4	TiO_2_ nanowire	NA	7.9	365	0.05	50	0.41	100	5.61E−07	1.12E−08	[74]
5	TiO_2–x_‐OV	NA	125	365	0.06	100	1	51	4.96E−08	4.96E‐10	[75]
6	KT‐0.1	20	0.13	352	0.3	10	300	58.4	3.12E−02	3.12E−03	This study

NA: Data not available.

## 3 Conclusion

This study systematically investigated the ability of KT‐x to remove FA from indoor air through PCO under various experimental conditions. A KOH modification enhanced the optical properties and photocatalytic activity of TiO_2_, leading to improved X_FA_. The surface and optical properties of KT‐x improved at lower doses of KOH due to the aggregation of potassium ions on the TiO_2_ surface. Raman spectroscopy analysis confirmed uniform structural modifications across all KT‐x samples, suggesting potassium‐induced alterations in the TiO₂ crystal lattice. These changes may play a crucial role in influencing their catalytic properties. Among all KT‐x samples, KT‐0.1 exhibited superior adsorption/decomposition performance of FA among the tested photocatalysts, achieving 100% X_FA_ for 100 ppm FA under 0.13 W of UV irradiation. Additionally, in the photocatalytic test under visible light, X_FA_ showed a significant reduction, averaging 25.2% lower than under UV irradiation. This decline may come from the lower photon energy of visible light, which may lead to rapid electron‐hole recombination with the reduced charge carrier excitation. KT‐0.1 also exhibited good stability when tested over four consecutive cycles. The FA PCO was also observed to be promoted under moderate RH levels. In situ, DRIFTS analysis revealed the FA PCO pathway and the roles of ROS in FA decomposition. Theoretical calculations revealed that the FA conversion mechanism is primarily driven by potassium and K─OH species, highlighting the critical roles of oxygen vacancies and potassium ions in enhancing the photocatalytic performance of KT‐x. Overall, KT‐x proves to be a highly effective candidate for developing a sustainable remediation system for VOCs in the air.

## 4 Experimental Section

### Materials and Chemicals

Only analytical‐grade chemicals were used in this study, and no further purification was performed unless specified otherwise. Commercial Degussa TiO_2_ (P25) was supplied by Evonik industries (Essen, Germany). Methanol (≥99.5%), KOH (≥85%), and paraformaldehyde (PFA, 95% OH(CH_2_O)_n_H) were supplied by Sigma–Aldrich (St. Louis, Missouri, USA). Ceramic beads (CBs) 1 mm in diameter were supplied by Nikkato Corp. (Osaka, Japan).

### KOH/TiO_2_ Synthesis

TiO_2_ was added to a 250 mL laboratory bottle containing 100 mL of an aqueous solution of KOH of varying molarities (0.1, 0.5, 1, and 2 mol L^−1^). The suspension was vigorously stirred for 300 min at 80 °C. Centrifugation was performed to recover the powder, which was then washed thoroughly with deionized H_2_O. A convection oven was used to dry the obtained powder at 80 °C for 24 h. The resulting photocatalyst was designated KT‐x, where x represents the alkali molar concentration used to synthesize KOH/TiO_2_. The photocatalysts were dip‐coated onto CBs, which were then washed ultrasonically with methanol for 120 min and heated at 100 °C (12 h). Next, 3 g of washed CBs and 30 mg of the photocatalyst were ultrasonicated in methanol for 3 h. Ball‐milling was performed to coat 10 mg of photocatalyst on the CB.

### Photocatalysis Experiments

Photocatalysis experiments were carried out using a laboratory‐scale packed‐bed continuous‐flow reactor comprising four UV‐A lamps (8 W and λ_max_ = 352 nm) and a quartz‐tube water jacket. However, the actual power of the UV‐A lamp reaching the catalyst surface was 0.13 W. To accurately assess photocatalytic efficiency, a performance evaluation based on photon flux as measured by quantum yield (QY) was conducted using 0.13 W. In addition, the water jacket was used to conduct the FA PCO reaction at room temperature (RT). A 20 L polyester aluminum (PEA; Top Trading Eng., Seoul, Republic of Korea) bag containing FA in the air was connected to the inlet of the reactor. A T junction was used to connect the reactor outlet to a large‐volume injector (LVI) and mini pump (MP‐Σ30KN II, Shibata Co., Ltd., Tokyo, Japan). The concentration of FA exiting the reactor ([FA_out_]) was monitored continuously using a gas chromatograph (GC)‐flame ionization detector (FID) (GC‐2030, Shimadzu Corp., Kyoto, Japan). The reactor effluent was sampled at 5 min intervals by the LVI to quantify the CO_2_ using a methanizer interfaced with the LVI‐GC‐FID. The measurements of carbon monoxide (CO) and CO_2_ were not conducted separately using the mechanized‐FID, as it measured carbon oxides (CO_x_) collectively. However, given the use of excess molecular oxygen (O_2_, 21 vol.%) in this study, the CO_x_ in the effluent was assumed to primarily consist of CO_2_. Additionally, no characteristic absorption signals for CO (2000–2100 cm^−1^) were detected in the in situ diffuse‐reflectance infrared Fourier‐transform spectroscopy (DRIFTS) spectra during FA oxidation, supporting the assumption (see Section 3.5).

The PFA was thermally degraded to produce a gaseous primary standard (G‐PS) of FA.^[^ ^62^ ^]^ In brief, 200 mg of PFA was loaded into a quartz tube with quartz wool end plugs. The G‐PS was produced by heating the PFA tube to 100 °C with an electric tube heater (TC200P, Misung Scientific Co. Ltd., Republic of Korea) under a nitrogen (N_2_) flow rate of 100 mL min^−1^ for 50 min. The resulting G‐PS was collected in a 5 L PEA bag. The concentration of the G‐PS was determined using standard 2,4‐dinitrophenylhydrazine high‐performance liquid chromatography (Nexera XR, Shimadzu Corp., Kyoto, Japan).

In situ, DRIFTS using a Nicolet iS50 FTIR spectrometer (Thermo Fisher Scientific, Waltham, MA, USA) equipped with an in situ diffuse‐reflectance cell (Praying Mantis, Harrick Scientific Products Inc., Pleasantville, New York, USA) was conducted to reveal the FA oxidation pathways. The DRIFTS analysis involved 32 scans at a resolution of 4 cm^−1^. Spectra were recorded for 50 mg of photocatalyst under various conditions at RT (e.g., dark, UV, and UV combined with moisture at a relative humidity of 20%). FA oxidation reactions were carried out using 100 ppm of FA in the air under continuous‐flow conditions for 1 h, with a volumetric flow rate (Q) of 100 mL min^−1^.

### Characterization and Instrumentation

The crystal structures of the photocatalysts were determined using powder X‐ray diffraction (PXRD: D8 ADVANCE, Bruker Corp., Billerica, Massachusetts, USA) with Cu Kα radiation (2θ 5–80°) source. Scanning electron microscope (SEM) images were collected using a Sigma 300 instrument (Carl Zeiss AG, Oberkochen, Germany). The textural properties of the photocatalysts were evaluated using N_2_ adsorption‐desorption isotherms acquired at 77 K through a Micromeritics 3Flex adsorption analyzer (Micromeritics Instrument Corp., Norcross, Georgia, USA). The surface chemistry of the tested photocatalysts was determined using K‐alpha X‐ray photoelectron spectroscopy (XPS, Thermo Fisher Scientific, Waltham, Massachusetts, USA). Adventitious carbon‐binding energy (285 eV) was used to charge‐correct the XPS data. Raman spectroscopy was performed at RT using a DXR3xi spectrometer (Thermo Fisher Scientific, Waltham, Massachusetts, USA) with a 1 mW laser operating at a wavelength of 532 nm. UV–vis light diffuse‐reflectance spectroscopy (DRS) was conducted with a Cary 5000 UV–vis‐near‐infrared spectrophotometer (Agilent Technologies, Inc., Santa Clara, California, USA). Photoluminescence spectra of the photocatalysts were obtained with an FP‐8200 spectrofluorometer (Jasco International Co., Ltd., Tokyo, Japan). The photoelectrochemical properties (Mott–Schottky [MS] analysis, photocurrent [PC] spectroscopy, and electrochemical impedance spectroscopy [EIS]) were investigated using a VersaSTAT4 electrochemical workstation (AMETEK Inc., Berwyn, Pennsylvania, USA). Photoelectrochemical analysis was conducted using a three‐electrode electrochemical cell filled with 0.5 m sodium sulfate (Na_2_SO_4_) electrolyte solution. The PEC setup comprised i) a working electrode (photocatalyst‐coated fluorine‐doped tin oxide conductive glass (2 × 2 cm working area), ii) a reference silver/silver chloride electrode, and iii) a platinum‐wire counter electrode. A 10 W UV light‐emitting diode was used for illumination. PC curves were recorded at 1 V versus a saturated calomel electrode (SCE). The EIS measurements were also conducted at 1 V versus SCE over a frequency range of 0.01 Hz to 100 kHz. Electron spin resonance (ESR) analysis was conducted using a 500 W UV light on a Bruker EMXnano spectrometer to investigate the generation of reactive oxygen species (ROS), including ^•^OH and ^•^O_2_^−^. A trapping agent of 5,5‐dimethyl‐1‐pyrroline N‐oxide (DMPO) was used to identify the ROS.

### Data Analysis

The photocatalytic activity was assessed quantitatively using FA removal efficiency (X_FA_ (%); Equation 4), QY (molecules photon^−1^; Equation 5), space‐time yield (STY in molecules photon^−1^ mg^−1^; Equation 6), and dynamic clean air delivery rate (DCADR in L mg^−1^ min^−1^; Equation 7).^[^ ^21^, ^63^, ^64^ ^]^ [FA_in_] represents the concentration of FA in the influent. QY is the ratio between the emitted photons and the absorbed photons on the surface of the photocatalyst. STY normalizes the QY per unit photocatalyst mass (m_cat_) used in the experiment. The DCADR measures the volume of clean air delivered per unit of time considering Q and the difference between [FA_in_] and [FA_out_].

(4) $$X_{FA}=\frac{\left[FA_{in}\right]-\left[FA_{out}\right]}{\left[FA_{in}\right]}\times 100$$

(5) $$\mathrm{QY}\;\left(\mathrm{molcules}\;\mathrm{photo}n^{-1}\right)=\frac{Decayrate\left(moleculessecond^{-1}\right)}{Photonflux\left(photonssecond^{-1}\right)}$$

(6) $$\mathrm{STY}\;\left(\mathrm{molecules}\;\mathrm{photo}n^{-1}g^{-1}\right)=\frac{QY\left(moleculesphoton^{-1}\right)}{m_{cat}\left(g\right)}$$

(7) $$DCADR=\frac{Q\left(Lmin^{-1}\right)\left[1-\frac{C_{out}}{C_{in}}\right]}{m_{cat}\left(g\right)}$$

The CO_2_ yield (Y_CO2_) was determined using Equation 8, where [CO_2_] denotes the concentration of CO_2_ in the reactor effluent. The relative standard error (RSE) was the critical quality assurance parameter for FA and CO_2_ quantification using the GC‐FID system. The RSE values for CO_2_ and FA were 0.88% and 0.57%, respectively. A fixed standard volume approach was employed using five standard concentrations of FA and CO_2_ (10, 20, 50, 100, and 200 ppm) to obtain calibration curves for the GC‐FID system. The calibration factors for FA and CO_2_ were 20.9 and 2445, respectively, with high coefficients of determination (R^2^) of 0.999 and 0.995. All experiments were conducted in duplicate, and the mean values were shown with error bars representing standard errors.

(8) $$Y_{CO_{2}}\left(\%\right)=\frac{\left[CO_{2}\right]\left(ppm\right)}{\left[FA_{in}\right]\left(ppm\right)}\times 100$$

### Density Functional Theory Model and Method

The pseudopotential method–based Spanish Initiative for Electronic Simulations with Thousands of Atoms code was used for the DFT simulations.^[^ ^65^ ^]^ All computations were performed using the generalized gradient approximation with Perdew–Burke–Ernzerhof functionals. Spin‐polarization and van der Waals corrections were used for weak non‐covalent interactions.^[^ ^66^, ^67^ ^]^ Norm‐conserving pseudopotentials for the core electrons were used to calculate the electronic ground state while optimizing the atomic locations. The wave function for all species other than hydrogen was expanded using the double‐ζ plus polarization basis of localized orbitals. In contrast, the double‐ζ basis was used to expand the hydrogen wave function (Troullier and Martins, 1991). The potassium, oxygen, titanium, carbon, and hydrogen cut‐off radii were 2.38, 1.47, 2.42, 1.14, and 1.25 au, respectively. The free energies of physical adsorption were estimated by the relationship ΔG = ΔH + ΔST, where ΔG represents the Gibbs free energy, and ΔH is the physisorption enthalpy (ΔH = (E_host+guest_ − E_host_ − E_guests_)/N_guests_).^[^ ^68^, ^69^ ^]^ Here, E refers to the net energy of the host system (prior to and following the guest molecule adsorption) + N_guest_ molecules in their isolated state. The change in entropy was estimated by the equation ΔS = H_vaporization_/T_vaporization_.^[^ ^70^ ^]^ An opposite sign was applied to adsorption‐free energy to calculate the desorption energy. The energy difference between the reactants and products was used to estimate the energy requirement for a given FA transformation step.

## Conflict of Interest

The authors declare no conflict of interest.

## Supporting information



Supporting Information

## Data Availability

The data that support the findings of this study are available from the corresponding author upon reasonable request.
