# Supplementary material for: The Photocatalytic Efficacy of Potassium Hydroxide–Based Modification of Titanium Dioxide in the Oxidative Destruction of Gaseous Formaldehyde
Source: Small. 2025 Apr 17;21(21):2501387. doi: 10.1002/smll.202501387 (PMC12105446; doi:10.1002/smll.202501387)
Supplement: Supplementary file 1 — Supporting Information [file SMLL-21-2501387-s001.docx]

**Supplementary Information (SI)**

**Table S1.** Physical properties of the tested photocatalysts.

| **Order** | **Photocatalyst** | **Average crystallite size (nm)** | | **BET surface area**  **(m^2^ g^-1^)** | **Pore volume**  **(cm^3^ g^-1^)** | **Average pore diameter**  **(nm)** |
| --- | --- | --- | --- | --- | --- | --- |
|  |  | **Anatase** | **Rutile** |  |  |  |
| 1 | TiO_2_ | 22.43 | 31.15 | 50.9 | 0.068 | 4.44 |
| 2 | KT-0.1 | 22.21 | 30.39 | 50.5 | 0.077 | 4.99 |
| 3 | KT-0.5 | 21.82 | 29.85 | 49.6 | 0.076 | 4.84 |
| 4 | KT-1 | 21.51 | 28.98 | 49.1 | 0.063 | 4.72 |
| 5 | KT-2 | 21.47 | 28.81 | 38.8 | 0.049 | 4.31 |

**Table S2.** XPS analysis of KT-x and TiO_2_ based on survey spectra.

| **Sample** | **Element** | | | |
| --- | --- | --- | --- | --- |
|  | **(atomic %)** | | | |
|  | **K 2p** | **C 1s** | **Ti 2p** | **O 1s** |
| KT-0.1 | 1.85 | 46.6 | 13.1 | 38.5 |
| KT-0.5 | 3.29 | 43.8 | 13.3 | 39.6 |
| KT-1 | 6.67 | 38.5 | 12.2 | 42.6 |
| KT-2 | 9.05 | 42.9 | 8.4 | 39.7 |
| TiO_2_ | 0 | 40.6 | 16.4 | 43.0 |

**
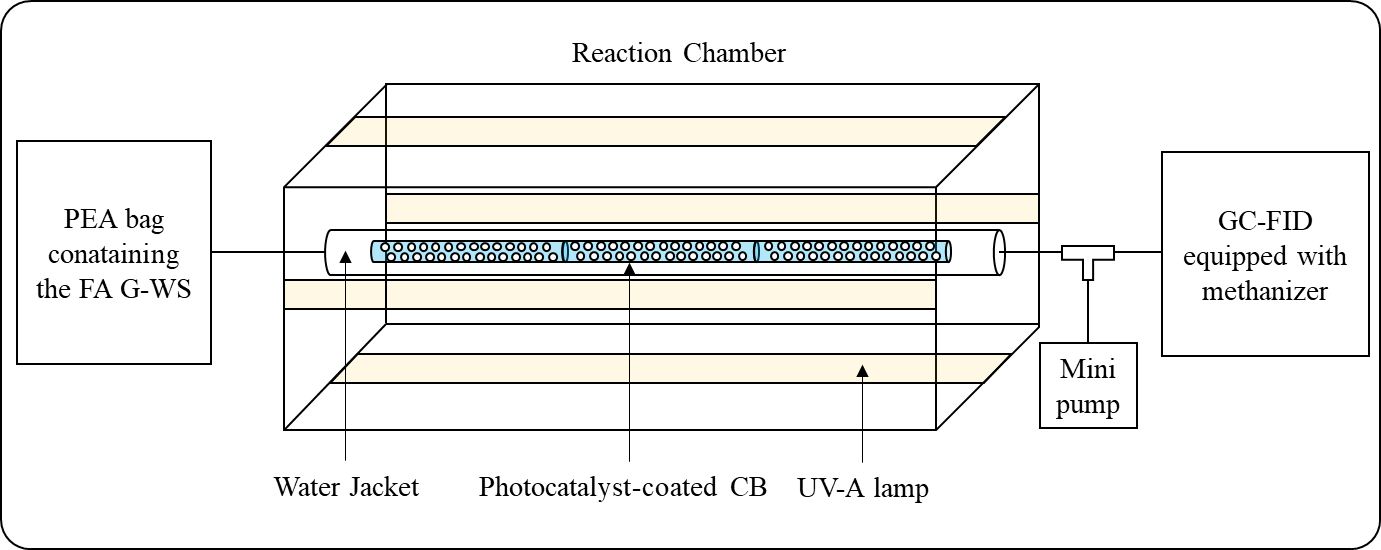
**

**Figure S1.** Schematic of the FA PCO experimental system.

**Figure S2.** FA removal performance of KT-3 and KT-5 as a function of time-on-stream ([FA_in_]: 100 ppm in air, Q: 100 mL min^-1^, RH: 0%, m_cat_: 10 mg, and light source: UV-A).

**Figure S3.** Comparison of photocatalytic performance of KT-x and TiO_2_ under UV and visible light illumination ([FA_in_]: 100 ppm in air, Q: 100 mL min^-1^, RH: 0%, and m_cat_: 10 mg).

**Figure S4.** Relative change in intensity of the primary *in* *situ* DRIFTS bands over time under UV irradiation for FA oxidation on the KT-0.1 and TiO_2_ surfaces: (a) OH, (b) DOM, and (c) formate.


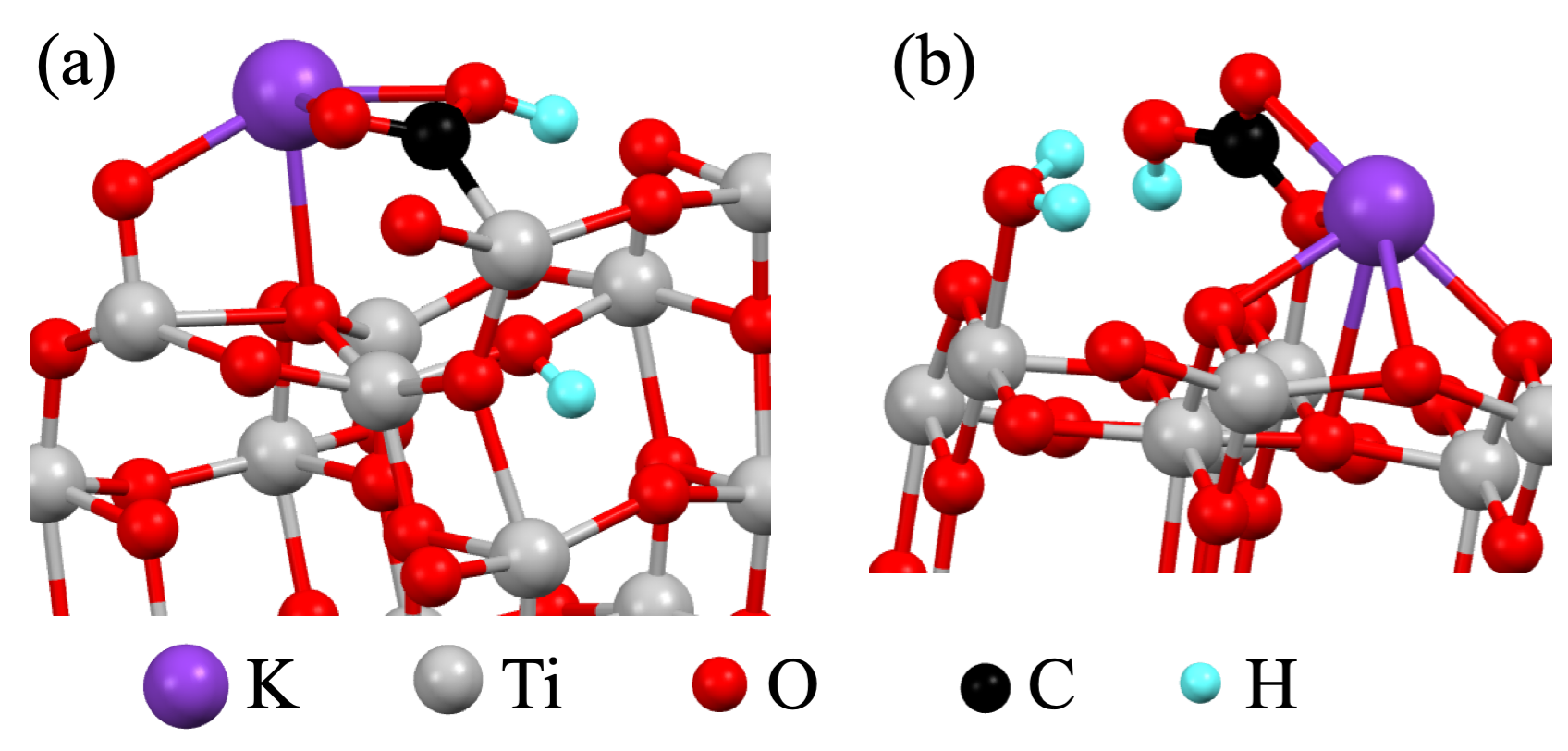


**Figure S5.** Optimized atomic structure in the final step of FA conversion over the (101) surface of TiO_2_: (a) potassium atom near an OV and (b) K-OH near an OV.
